# Interaction of mumps virus V protein variants with STAT1-STAT2 heterodimer: experimental and theoretical studies

**DOI:** 10.1186/1743-422X-7-263

**Published:** 2010-10-11

**Authors:** Nora H Rosas-Murrieta, Irma Herrera-Camacho, Helen Palma-Ocampo, Gerardo Santos-López, Julio Reyes-Leyva

**Affiliations:** 1 Laboratorio de Bioquímica y Biología Molecular, Instituto de Ciencias, Benemérita Universidad Autónoma de Puebla. Edif. 103 H, CU-BUAP, San Manuel, CP 72550, Puebla. México; 2 Laboratorio de Virología, Centro de Investigación Biomédica de Oriente, Instituto Mexicano del Seguro Social, Km 4.5 carretera Atlixco-Metepec, CP 74360 Metepec, Puebla, México

## Abstract

**Background:**

Mumps virus V protein has the ability to inhibit the interferon-mediated antiviral response by inducing degradation of STAT proteins. Two virus variants purified from Urabe AM9 mumps virus vaccine differ in their replication and transcription efficiency in cells primed with interferon. Virus susceptibility to IFN was associated with insertion of a non-coded glycine at position 156 in the V protein (VGly) of one virus variant, whereas resistance to IFN was associated with preservation of wild-type phenotype in the V protein (VWT) of the other variant.

**Results:**

VWT and VGly variants of mumps virus were cloned and sequenced from Urabe AM9 vaccine strain. VGly differs from VWT protein because it possesses an amino acid change Gln_103_Pro (Pro^103^) and the Gly^156 ^insertion. The effect of V protein variants on components of the interferon-stimulated gene factor 3 (ISGF3), STAT1 and STAT2 proteins were experimentally tested in cervical carcinoma cell lines. Expression of VWT protein decreased STAT1 phosphorylation, whereas VGly had no inhibitory effect on either STAT1 or STAT2 phosphorylation. For theoretical analysis of the interaction between V proteins and STAT proteins, 3D structural models of VWT and VGly were predicted by comparing with simian virus 5 (SV5) V protein structure in complex with STAT1-STAT2 heterodimer. *In silico *analysis showed that VWT-STAT1-STAT2 complex occurs through the V protein Trp-motif (W^174^, W^178^, W^189^) and Glu^95 ^residue close to the Arg^409 ^and Lys^415 ^of the nuclear localization signal (NLS) of STAT2, leaving exposed STAT1 Lys residues (K^85^, K^87^, K^296^, K^413^, K^525^, K^679^, K^685^), which are susceptible to proteasome degradation. In contrast, the interaction between VGly and STAT1-STAT2 heterodimer occurs in a region far from the NLS of STAT2 without blocking of Lys residues in both STAT1 and STAT2.

**Conclusions:**

Our results suggest that VWT protein of Urabe AM9 strain of mumps virus may be more efficient than VGly to inactivate both the IFN signaling pathway and antiviral response due to differences in their finest molecular interaction with STAT proteins.

## Background

Interferon induces the major defense against viral infections. It begins with attachment of IFN-α or -β to heterodimeric receptors composed of IFNAR1 and IFNAR2 subunits whose intracellular domains are associated with Tyk2 and Jak1 tyrosine kinases, respectively [1]. Activation of the signal transduction occurs when Tyk2 phosphorylates Tyr^466 ^residue on IFNAR1, creating a docking site for STAT2 that is phosphorylated on Tyr^690^. Phosphorylated STAT2 protein then associates with STAT2, inducing its phosphorylation on Tyr701 by JAK1 [2,3]. STAT1 and STAT2 form a heterodimer that creates a nuclear localization signal (NLS). STAT1-STAT2 heterodimers result from intermolecular interactions between Src homology 2 (SH2) domains and phosphorylated Tyr residues at each protein [4]. In addition, IFNAR2 subunit is acetylated at Lys^399 ^and promotes the acetylation of IRF9, which is essential to DNA binding [5,6]. Association of STAT1-STAT2 heterodimer with IRF9 constitutes the IFN-stimulated gene factor 3 (ISGF3) transcription factor, which binds to IFN-stimulated response elements (ISRE) at IFN-stimulated genes (ISG). The final step of this signaling pathway is the induction of gene transcription whose expression establishes the antiviral state [2,7]. Several viruses have evolved strategies to circumvent the antiviral state stimulated by IFN through the expression of proteins that antagonize some components of the IFN signaling pathway such as the V protein of paramyxoviruses [8]. Mumps virus P gene codes for three polypeptides: V, I and P. Their mRNAs are translated by use of overlapping reading frames (ORFs) via cotranscriptional insertion of nontemplated guanidine nucleotides (mRNA edition) [9,10]. Mumps virus V protein is a nonstructural protein that counteracts the IFN-induced antiviral response [11].

Paramyxovirus V proteins possess an identical N-terminal sequence with P and I proteins but have a unique C-terminal that contains two functional motifs [9]. The first is the cysteine-rich (Cys-rich) motif (CX3CX11CXCX2CX3CX2C) where × refers to any amino acid residue that establishes a stoichiometric relationship (1:2) with Zn^2+^. Cys-rich motif is highly conserved among rubulaviruses such as simian virus 5 (SV5), simian virus 41 (SV41), human parainfluenza virus type 2 (hPIV2), and mumps virus. Cys-rich motif promotes the formation of an oligomer that acts as a nucleation site known as V-dependent degradation complex (VDC) where both polyubiquitylation and degradation of STAT1 occur [12,13]. The V proteins of mumps virus and SV5 induce the degradation of STAT1 protein through the VDC assembly that includes ubiquitin ligase E3, Roc1, Cul4A, and DDB1 proteins that facilitate polyubiquitylation of STAT1 [13,14]. The second C-terminal motif is also involved in STAT1 degradation and is a Trp-motif (W-(X)3-W-(X)9-W) that includes W^174^, W^178 ^and W^188 ^residues located upstream of the Cys-rich motif [15,16]. The C-terminal of V protein is essential for successful viral infection by inhibition of IFN signaling and blocking of the antiviral response [17]. In this study we analyzed two variants of mumps virus V protein (VWT and VGly) derived from Urabe AM9 vaccine strain. Previous studies have shown that Urabe AM9 vaccine is constituted by several quasispecies that differ in distinct sites all along their genomes. We purified two virus variants based on the sequence of their HN gene and were named HN-A1081 and HN-G1081, which codes for HN-K335 and HN-E335 proteins, respectively. Several studies have related HN-A1081 with neurovirulence because this virus variant was frequently isolated from patients with postvaccine aseptic meningitis [18]. We demonstrated that HN-A1081 variant preferentially infects nerve cells, whereas HN-G1081 variant has limited replication in nerve cells. Selective infection of nerve cells was associated with differences in the virus binding affinity towards cell receptors [19]. However, further experiments showed that differences in sensitivity to IFN determined the replication rate of Urabe AM9 mumps virus variants in nerve cells. Indeed, HN-A1081 virus variant evaded the IFN-induced antiviral response and replicated in cells primed with IFN, whereas HN-G1081 variant reduced both replication and transcription in IFN-primed cells [20]. Sensitivity to IFN was associated with insertion of a non-coded glycine at position 156 in the V protein (VGly) of HN-G1081 virus variant, whereas resistance to IFN was associated with preservation of wild-type phenotype in the V protein (VWT) of HN-A1081 Virus variant. In the present study we experimentally tested the interaction of VWT and VGly proteins of Urabe AM9 mumps virus variants with proteins of the IFN signaling pathway, finding differences in their capacity to bind STAT proteins. In addition, *in silico *three- dimensional structure models of VWT and VGly proteins supported their difference to form complexes with STAT1 and STAT2 *in vitro*. The relevance of these theoretical findings in the function of V protein and virulence of mumps virus variants are discussed.

## Results

In order to determine the effect of protein V of the majority populations that comprise the Urabe AM9 vaccine strain on the IFN pathway, we obtained the coding region for V proteins from HN-A1081 and HN-G1081 virus variants, which were cloned in the pcDNA4/HisMax TOPO vector (pcDNA4/HisMaxVA and pcDNA4/HisMaxVG) to add a His-tag at the amino end. Next, the full sequence of V ORF was determined (675 bp), and *in silico *translation was carried out by comparative analysis. Amino acid differences between V proteins were determined by comparison with the V protein from Urabe AM9 (SmithKline Beecham) (Protein: AAK60067.1). The VA protein containing only 224 residues similar to the wild-type V protein type was named VWT (28.17 kDa). The VG protein contained two changes on residue 103, Q→P, and the addition of a glycine residue at position 156, which generated a V protein with 225 amino acids and was designated VGly (28.13 kDa). Comparing both V proteins, there were no significant changes in the theoretical physicochemical parameters.

**Figure 1:**
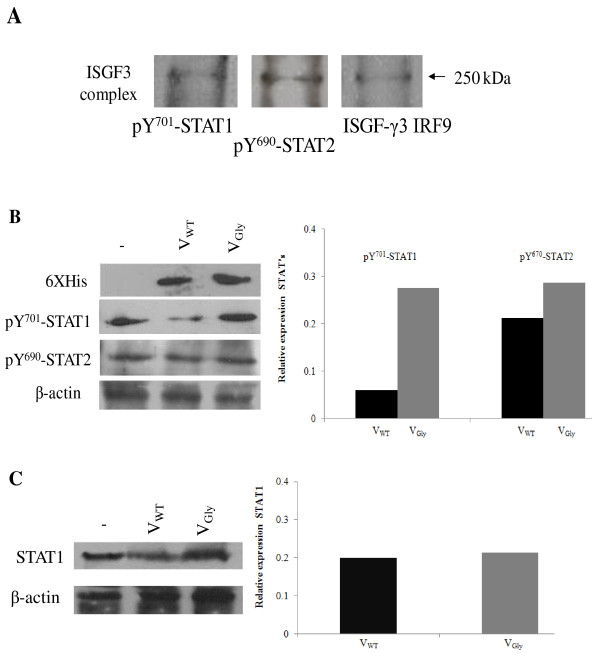
**Decrease in pY^701^-STAT-1 level protein by VWT of Urabe AM9 vaccine strain**. (A) Detection of ISGF3 complex activated by IFN-α in human cervical carcinoma cell line. The complex was determined 48 h after transfection and 6 h after stimulation with IFN proteins separated by 7% PAGE under native conditions, semidry transfer to PVDF membrane and immunodetection with specific antibodies for pTyr^701^-STAT1 proteins, pTyr^690^-STAT2 and IRF9 (ISGF-γ3). The molecular weight of the complex is 250 kDa. (B) Effect of VWT and VGly proteins on STAT1 and STAT2 phosphorylated proteins by Western blot in human cervical carcinoma cell line. Proteins were separated in 10% SDSPAGE with semidry transfer to PVDF membrane and immunodetection with antibodies against His-tag for V proteins, pTyr^701^-STAT1, pTyr^690^-STAT2 and β-actin. (C) Detection of STAT1 inactive protein in cell expressing VWT and VGly proteins and 10% SDS-PAGE transfer to PVDF membrane and immunodetection with antibodies against STAT1 inactive protein and β-actin to normalize the level protein.

To determine the effect of VWT and VGly proteins on the IFN pathway, they were expressed in cervical carcinoma cells stimulated with IFN-α2b. First we determined the ISGF3 complex formation in response to IFN-α2b by detecting STAT1, STAT2 and IRF9 proteins in cells stimulated with IFN and the proteasome inhibitor MG132. Figure 1A shows the ISGF3 complex of 250 kDa identified with the three antibodies used. This result indicates the ability of the cells to activate the antiviral IFN pathway. Next we examined the effect of V protein on the level of Y^701^-STAT1 and Y^690^-STAT2 phosphorylated proteins. Figure 1B demonstrates that cells expressing protein VWT decreased phosphorylated STAT1 protein.

None of the variants changed the level of active STAT2 protein as determined with other strains of mumps virus. To test whether the result in Figure 1B was due only to reduction of active STAT1 protein or by degradation of STAT1 unphosphorylated protein, we studied the level of inactive STAT1. Figure 1C shows that in cells expressing VWT and VGly proteins there are no changes in the level of STAT1 protein. This suggests that VWT protein of Urabe AM9 affects the STAT1 phosphorylated protein in blocking type I IFN system. In other strains of mumps virus and SV5, reduction of the STAT1 protein was always determined in the heterodimer with STAT2 phosphorylated protein with the subsequent blockade of the IFN system [21]. Figure 1B, C suggests a differential effect of the V proteins of Urabe AM9 strain vaccine on antiviral cellular response, which may be due to different interactions of VWT and VGly proteins with STAT1-STAT2 heterodimer.

**Figure 2:**
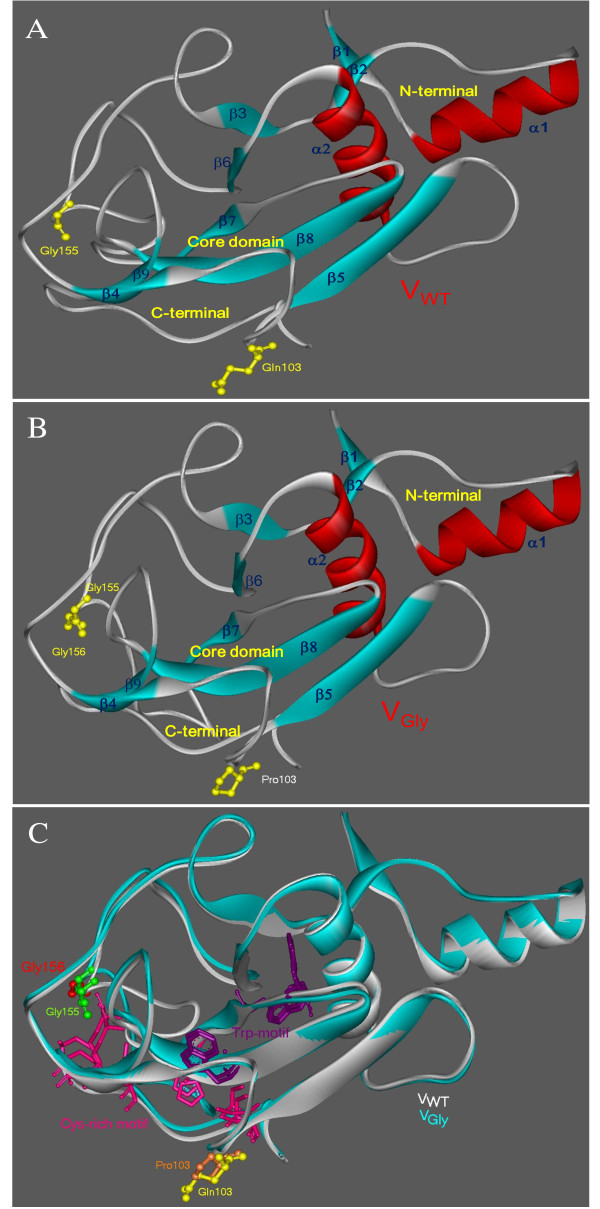
**Homologous modeling and differences of theoretical 3D structure of VWT and VGly**. (A) Models of V proteins, VWT and (B) VGly built with the PDB: 2B5Lc as template. Both cases show residues 155 and 103. Additionally, residue 156 is shown in B. (C) Superimposition of the 3D models of VWT (gray) and VGly (blue), Gly^155 ^(green), Gly^156 ^of VGly (red), Pro^103 ^of VGly (orange), Gln^103 ^of VWT (yellow), Trp-motif residues of binding to STAT1-STAT2 (purple), Cys-rich motif C4HC3 (residues in pink). Display in Web Lab Viewer.

To analyze this assumption we studied the theoretical interaction between VWT and VGly proteins and IFN pathway proteins. Theoretical 3D structure prediction of VWT and VGly was first performed by homology using the tertiary structure of V-SV5, PDB: 2B5Lc[22], which lack three loops in positions 1-15, 55-80 and 153-159. The identity of VWT and VGly with the template was 39%. The 3D model of VWT originates in amino acid 37 and ends in 220 (183 residues), whereas the VGly model originates in position 37 and ends in 221 (184 residues). Qualitative values of the 3D models were in the expected region for structural models of proteins with values of PROSA Z-score as follows: -2.71, -2.35 and -2.47 for VWT, VGly and V-SV5, respectively, used as template protein. Theoretical 3D structure of VWT and VGly proteins can be described as an N-terminal domain, which adopts an α-helical structure (α1), a core domain with a central seven-stranded β sheet rounded by an α helix (α2) and two loops in the C-terminal end (Figure 2A, 2B). To observe the differences between 3D models in both proteins, we performed a superimposition of structures. Figure 2C shows the theoretical changes in the 3D models. In VGly there is an arrangement of loops connecting β3 and β4 strands such as the loop between β6 and β7 where Gly^156 ^was inserted, although the most evident modification is the subsequent region to the Gly insertion where the Cys-rich motif is located (amino acids in pink, Figure 2C). The presence of Pro^103 ^in VGly (residues in orange and yellow in Figure 2C) does not significantly modify the theoretical structure of the V protein. All residues of the Trp-motif were modified in regard to Trp-motif in VWT (amino acids in purple, Figure 2C).

**Figure 3:**
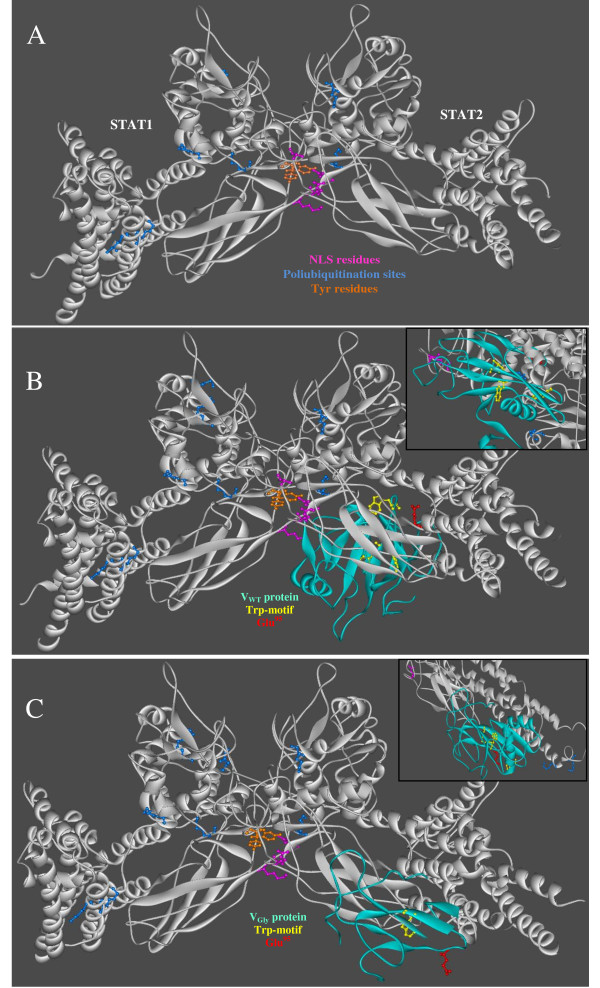
**Interaction model V-STAT1-STAT2**. (A) Heterodimer model of STAT1-STAT2 by the SH2-domain (STAT1 PDB: 1YVL and 3D model of STAT2 from 1YVL and 1BF5). Heterodimer model shows the following residues: Tyr^690 ^and Tyr^701 ^(orange), nuclear localization signal residues (pink), lysine residues to ubiquitylation (Ub) (blue). (B) Interaction of STATs heterodimer with VWT by Trp-motif and STAT2. (C) Interaction of heterodimer with VGly by STAT2, lysine residues (Ub) (blue). In B and C, the boxes at right show the interaction site.

For the formation of STAT1-STAT2 heterodimer activated by IFN, the 3D structure of STAT1 was obtained from PDB: 1YVL (structure of unphosphorylated STAT1) [23] and the 3D theoretical model of STAT2 by homology from templates PDB: 1BF5 (tyrosine phosphorylated STAT-1/DNA complex) [24] and PDB: 1YVL with the purpose of obtaining the 3D model that includes Tyr^690 ^required for interaction with STAT1 (identity was 46% with STAT1). According to the analysis, the site of interaction on the receptor (STAT2) was set in positions 690 and 698 and the ligand binding site (STAT1) was set in positions 701 and 708, which included the amino acids Tyr^690 ^and Tyr^701 ^of STAT2 and STAT1, respectively, to achieve formation of the dimer by interaction of their SH2-domains (573- 670 in STAT1 and 572-667 in STAT2). The model of STAT1-STAT2 dimer corresponded to the solution of lowest overall energy (-43.71) with attractive and repulsive van der Waals energy of -29.86 and 14.05, respectively; an atomic contact energy of -5.27 and an energy of -3.35 derived from formation of hydrogen bonds. Construction of this model was based on the phosphorylated STAT1 model [24]. The following were located in the heterodimer model (Figure 3A): residues of Tyr^690 ^and Tyr^701 ^(orange) and NLS residues in STAT1: Lys^410 ^and Lys^413^, in STAT2 Arg^409 ^and Lys^415 ^(pink), potential ubiquitylation sites in STAT1 (K^85^, K^87^, K^296^, K^413^, K^525^, K^679^, K^685^) and STAT2 (K^178^, K^182^, K^543^, K^681^) (blue). Next we analyzed the model of interaction between V proteins and STAT proteins. The VWT-STATs complex had an overall binding energy of -57.08 with atomic contact energy of -1.43, attractive and repulsive van der Waals energy of -68.32 and 34.70, respectively, and energy of -4.38 derived from the formation of hydrogen bonds. In the interaction model of VWT-STATs complex, that interaction occurs through STAT2 near Arg^409 ^and Lys^415 ^of NLS without interference from amino acids Lys^410 ^and Lys^413 ^in NLS of STAT1 (Figure 3B, checkbox). The analysis showed that the interaction occurs through the Trp-motif and Glu^95 ^(residue equivalent to Asn^100 ^of V-SV5 that interacts with STAT2). In V-SV5, the change from Asn^100^→Asp^100 ^maintained the ability of interaction with STAT2 [25]. In mumps virus V protein, the relatively conservative conversion of glutamic acid to an aspartic acid (E95D) resulted in a V protein still capable of blocking STAT1 signaling [26]. Several studies demonstrated that the mumps virus V protein requires STAT2 to promote the degradation of STAT1 through the proteasome [13,21,27]. We hypothesize that the association of V with STAT2 would leave STAT1 susceptible to ubiquitination. The seven potential ubiquitylation sites in STAT1 would not be blocked by the association with VWT. The model VGly-STATs complex had an overall energy of interaction of -92.74, atomic contact energy of -11.21, attractive and repulsive van der Waals energy of -54.45 and 22.00, respectively, and energy of -4.24 derived from the formation of hydrogen bonds. In the theoretical model of VGly-STATs complex, the interaction occurs through STAT2 but far from the NLS of STAT1 and STAT2 (Figure 3C, box). However, the contact among proteins does not occur due to the Trp-motif or Glu^95 ^(Figure 3C). On the other hand, the interaction of VGly with the heterodimer does not prevent ubiquitylation of the lysine residues of both STAT1 and STAT2, although two Lys amino acids (178 and 182) are near the interaction site of VGly with STAT2.

## Discussion

The lack of antiviral for specific control of mumps virus infection requires the study of the molecular mechanism of replication and viral expression to propose sites related to the blocking of viral infection. The Urabe AM9 mumps vaccine is associated with virulence and is composed of at least two viral variants [18,28,29]. HN-A1081 variant selectively and preferentially infects nerve cells, whereas HN-G1081 has limited replication in these cells. It is interesting to explore the differences of the potential determinants of a successful viral infection in the nervous system [18,19]. Considering that V protein of the *Paramyxoviridae *family is a factor that facilitates viral replication by blocking certain steps in the IFN pathway, there may be a difference between V proteins from Urabe AM9. We currently know that the V protein from wild-type mumps virus, Torri and Enders strains, is associated with STAT1-STAT2 to prevent antiviral cellular response [11,13,30,31].

In this study we analyzed both *in vitro *and *in silico *two variants of V protein Urabe AM9: VWT (related to aseptic meningitis) and VGly. Amino acid sequence analysis showed that VGly is different from VWT at Pro^103 ^and Gly^156^. Such changes altered the theoretical 3D structure and possibly its anti-IFN function. The analysis of the effect of the V protein on STATs proteins showed the efficiency of VWT protein to promote the reduction of STAT1 active protein, whereas VGly protein did not affect its level. This fact has been demonstrated in others strains [13,21,26,31]. Such data suggest that the structural changes on VGly induced by rearrangement of loops and residues of the Cys-rich and Trp-motifs following the addition of Gly^156 ^may be responsible for the loss of efficiency in inducing degradation of the STAT1 protein. This could explain the differences reported in the replication and transcription of genes in response to interferon during infection with the variant HN-G1081 (VGly) of Urabe AM9 where the induction of genes in response to interferon is higher than in the presence of an infection with the variant HN-A1081 (VWT) [20]. However, we cannot conclude if the changes induced by the addition of Gly^156 ^and the low efficiency in the degradation of STAT1 protein for the variant VGly are conferred by inefficient interaction with the proteins involved in the ubiquitylation and degradation by the proteasome system (E2, DDB1, Cullin, Roc1) [14]. These must be confirmed experimentally. To outline a likely explanation, it was predicted the theoretical 3D structure of VWT and VGly by homology modeling. Although the 2B5Lc template lack three loops not resolved by X-ray diffraction, the program modeled two mobile loops but we cannot provide a conclusion of the modeled structure without the template for comparison. The changes mentioned in the VGly modified the theoretical 3D structure, particularly in the loops that limit the Cys-rich motif. The residues of these motifs in VGly move away from Gly^155 ^(present in both proteins), altering the 3D distribution. It is possible that residues in Cys and Trp motifs of VWT are those related to the activity anti-IFN of the V protein of Urabe AM9 mumps vaccine.

At the experimental level, the intermolecular interaction of mumps virus V protein and V-SV5 with the cellular protein of type I IFN by the VDC complex has been demonstrated: STAT1-STAT2 (both phosphorylated), DDB1, Cullin 4A and Roc1 [13]. Interestingly, the interaction of VWT occurs through STAT2, an area near NLS residues [32] that would prevent their importation to the nucleus by steric hindrance. The theoretical interaction with

STAT2 could maintain the heterodimer in the cytoplasm where the ubiquitin/proteasome labels the lysine-susceptible residues exposed in STAT1. *In vivo*, it has been shown that the promotion of degradation of STAT1 by the V protein of MuV and V-SV5 is dependent on

STAT2 in the VDC complex [13,14,21,26,33,34]. In any case it would block signal transduction of type I IFN to the nucleus, avoiding the antiviral cellular state favorable to viral replication of HN-A1081 variant of Urabe AM9. Instead, *in silico *analysis of the theoretical interaction between VGly and STAT1-STAT2 showed that the contact occurs through STAT2 as in VWT but in a region far from residues of the NLS on STAT1/STAT2. This would suggest that the heterodimer may advance to the nucleus for exercising its transcriptional activity, although the majority of lysine residues able to bind to ubiquitin are exposed. Although the comparison of the interaction parameters showed that the complex VGly with the STATs proteins may be more stable in terms of overall energy interaction, the attractive and repulsive van der Waals forces were higher in the complex between VWT and

STAT1-STAT2 proteins. The data obtained would explain the reduced capacity of VGly to block the IFN transduction signal, generating a cellular environment unfavorable for viral infection [27].

## Conclusions

The *in silico *analysis suggests that, *in vivo*, VWT may be more efficient than VGly to associate with the STATs proteins and probably for blocking the IFN transduction signal as a mechanism to avoid the antiviral defense.

## Methods

### Cell culture

The cervical carcinoma cell lines HeLa and C33A were used for transfections assays and were maintained in Dulbecco's minimum essential medium (Sigma, St. Louis, MO, USA) supplemented with 10% fetal bovine serum (Gibco-BRL, Grand Island, NY), 100 U/mL penicillin, 100 μg/mL streptomycin and 1% nonessential amino acids (Sigma, St. Louis, MO, USA). Cells were incubated at 37°C in 5% CO2.

### Subcloning of VA and VG ORF

The cloning of VA and VG ORF were performed by PCR from pCR-TOPO-VA and pCRTOPO-VG building in a previous work [20] with the oligonucleotides MuV-1 D 5'-GACCAATTTATAAAACAAGATGAGACTGGT-3' and MuV2 5'-TCCATCCCTCTAAGGAGGTCC-3' (IDT, Coralville, IA). PCR fragment was subcloned in the pCDNA4/HisMax TOPO vector (Invitrogen, Carlsbad, CA, USA) according to the manufacturer's instructions. This vector added a His-tag at the N-terminal of V proteins. Recombinant DNA was transformed in *E. coli *TOP 10 One Shot (Invitrogen, Carlsbad, CA, USA). Positive clones were sequenced by Big Dye ABI chemistry.

### Transient transfection assay and IFN treatment

A monolayer of adenocarcinome cervix cells grown to 80% confluence on flasks of 25 cm^2 ^was transfected with 6 μg of vector DNA (pCDNA4/His/Max-VA and VG) and TurboFect transfection reagent (Fermentas, Glen Burnie, MD, USA) according to the manufacturer's instructions. After cultivation for 24 h, the cells were stimulated with the proteasome inhibitor MG132 (40 μM) (Sigma, St. Louis, MO, USA). At 42 h after transfection, the cells were treated with 4000 IU/mL of IFN-α2b (Urifrón) (Probiomed, Mexico) for 6 h.

### Western Blot Analysis

After the stimulation with IFN-α2b and MG132, the cells were lysed with ProteoJET Mammalian Cell Lysis Reagent (Fermentas, Glen Burnie, MD, USA), and the cell lysates were lyophilized and solubilized by boiling for 10 min with sodium dodecyl sulfate (SDS)-polyacrylamide gel electrophoresis (PAGE) sample buffer (62.5 mM Tris-HCl pH 6.8, 5% 2-mercaptoethanol, 2% SDS, 0.005% bromophenol blue, 10% glycerol). The proteins were transferred to a PVDF membrane (0.45 μm) (Santa Cruz Biotechnology, Santa Cruz, CA, USA). The membrane was treated with primary antibody (p-Tyr^701 ^Stat1: sc-7988, p-Tyr^690 ^Stat2: sc-21689, ISGF-3 p48: sc-10793, Actin: sc-8432, His-probe: sc-8036 (Santa Cruz Biotechnology) for 1 h and then incubated with the secondary antibody (bovine antirabbit IgG-HRP: sc-2370) (Santa Cruz Biotechnology) for 1 h. After extensive washing, the immunoreactive bands were detected with Immobilon Chemiluminiscent substrate (Millipore Corporation, Bedford, MA, USA). For detection of the ISGF3 complex, proteins were separated by electrophoresis through 7.5% polyacrylamide gels, transferred to PDVF membranes, and detected with the previously mentioned antibodies.

### Generation and analysis of 3D protein models

The prediction for homology of the 3D protein structure was performed with the Swiss-Model program [35] using as template the structure of V protein simian virus 5 (V-SV5) at 2.85 Å by X-ray diffraction [22]. Neighboring protein structures of mumps virus V proteins were obtained with VAST search [36]. Theoretical 3D structure of VWT and VGly was visualized with Web Lab Viewer program. The final theoretical 3D structures were analyzed with PROCHEK of Swiss-Model [37,38] and with PROSA [39]. The theoretical 3D model of STAT2 was obtained for homology on Geno3D [40] from the PDB: 1BF5 (Tyrosine phosphorylated STAT-1/DNA complex) [24] and PDB: 1YVL. Electrostatic potential was obtained with the Poisson-Boltzmann method in Deep View from Swiss PDB Viewer. The differences between VWT and VGly were analyzed in the SuperPose program [41]. Polyubiquitylation sites in STATs proteins were predicted with UniPred [42], considering as probable those Lys residues with a minimum score of 0.7 to 1.

### Theoretical interaction

Theoretical heterodimer STAT1-STAT2 model was obtained by a docking analysis with Hex server [43]. The putative interaction models between VWT and VGly with STAT1-STAT2 proteins were generated with PatchDock server (Molecular Docking Algorithm Based on Shape Complementary Principles) [44] and 1000 theoretical models were refined on FireDock (Fast Interaction Refinement in Molecular Docking) [45].

## Competing interests

The authors declare that they have no competing interests.

## Authors' contributions

NHRM carried out the molecular techniques: nucleic acid purification, PCR, subcloning, transfection assays, and Western blot analysis and participated in the *in silico *sequence analysis and in drafting of the manuscript. IHC participated in sequence alignment and in data analysis. HPO participated in the subcloning, transfection assays and Western blot analysis. GSL participated in data analysis and helped to draft the manuscript. JRL participated in data analysis and helped to draft the manuscript. All authors read and approved the final manuscript.

## References

[B1] Goodbourn S Didcock L Randall RE Interferons: cell signaling, immune modulation, antiviral response and virus countermeasures J Gen Virol 2000 81 10 2341 2364 10993923 10.1099/0022-1317-81-10-2341

[B2] Randall RE Goodbourn S Interferons and viruses: an interplay between induction, signalling, antiviral responses and virus countermeasures J Gen Virol 2008 89 1 1 47 10.1099/vir.0.83391-0 18089727

[B3] de Weerd NA Samarajiwa SA Hertzog PJ Type I interferon receptors: biochemistry and biological functions J Biol Cel 2007 282 28 20053 20057 10.1074/jbc.R700006200 17502368

[B4] Samuel CE Antiviral actions of interferons Clin Microbiol Rev 2001 14 4 778 809 10.1128/CMR.14.4.778-809.2001 11585785 PMC89003

[B5] Banninger G Reich NC STAT2 nuclear trafficking J Biol Chem 2004 279 38 39199 39206 10.1074/jbc.M400815200 15175343

[B6] Tang X Gao JS Guan YJ McLane KE Yuan ZL Ramratnam B Chin YE Acetylation-dependent signal transduction for type I interferon receptor Cell 2007 131 1 93 105 10.1016/j.cell.2007.07.034 17923090

[B7] Murray PJ The JAK-STAT signaling pathway: input and output integration J Immunol 2007 178 5 2623 2629 17312100 10.4049/jimmunol.178.5.2623

[B8] Horvath CM Weapons of STAT destruction Eur J Biochem 2004 271 23-24 4621 4628 10.1111/j.1432-1033.2004.04425.x 15606749

[B9] Lamb RA Kolakofsky D Fields BN, Knipe DM, Howley PM, Griffin DE Paramyxoviridae: the viruses and their replication Fields Virology 2001 4 Philadelphia: Lippincott-Raven Publishers 1305 1340

[B10] Paterson RG Lamb RA RNA editing by G-nucleotide insertion in mumps virus P-gene mRNA transcripts J Virol 1990 64 9 4137 4145 2166809 10.1128/jvi.64.9.4137-4145.1990 PMC247877

[B11] Gotoh B Komatsu T Takeuchi K Yokoo J Paramyxovirus accessory proteins as interferon antagonists Microbiol Immunol 2001 45 12 787 800 11838896 10.1111/j.1348-0421.2001.tb01315.x

[B12] Kubota T Yokosawa N Yokota S Fujii N C terminal Cys-rich region of mumps virus structural V protein correlates with block of interferon α and γ signal transduction pathway through decrease of STAT1-α Biochem Biophys Res Commun 2001 283 1 255 259 10.1006/bbrc.2001.4764 11322797

[B13] Ulane MC Kentsis A Cruz CD Parisien JP Schneider KL Horvath CM Composition and assembly of STAT-targeting ubiquitin ligase complexes: paramyxovirus V protein carboxyl terminus is an oligomerization domain J Virol 2005 79 16 10180 10189 10.1128/JVI.79.16.10180-10189.2005 16051811 PMC1182666

[B14] Andrejeva J Poole E Young DF Goodbourn S Randall RE The p127 subunit (DDB1) of the UV-DNA damage repair binding protein is essential for the targeted degradation of STAT1 by the V protein of the paramyxovirus simian virus 5 J Virol 2002 76 22 11379 11386 10.1128/JVI.76.22.11379-11386.2002 12388698 PMC136798

[B15] Nishio M Garcin D Simonet V Kolakofsky D The carboxyl segment of the mumps virus V protein associates with Stat proteins in vitro via a tryptophan-rich motif Virology 2002 300 1 92 99 10.1006/viro.2002.1509 12202209

[B16] Nishio M Tsurudome M Ito M Garcin D Kolakofsky D Ito Y Identification of Paramyxovirus V protein residues essential for STAT protein degradation and promotion of virus replication J Virol 2005 79 13 8591 8601 10.1128/JVI.79.13.8591-8601.2005 15956600 PMC1143765

[B17] Sun M Rothermel TA Shuman L Aligo JA Xu S Lin Y Lamb RA He B Conserved cysteine-rich domain of paramyxovirus simian virus 5 V protein plays an important role in blocking apoptosis J Virol 2004 78 10 5068 5078 10.1128/JVI.78.10.5068-5078.2004 15113888 PMC400337

[B18] Santos-López G Cruz C Pazos N Vallejo V Reyes-Leyva J Tapia-Ramírez J Two clones obtained from Urabe AM9 mumps virus vaccine differ in their replicative efficiency in neuroblastoma cells Microbes Infect 2006 8 2 332 339 10.1016/j.micinf.2005.06.031 16298153

[B19] Reyes-Leyva J Baños R Borraz-Arguello M Santos-Lopez G Alvarado G Rosas N Herrera I Vallejo I Tapia-Ramírez J Amino acid change 335 E to K affects the sialic acid-binding affinity and neuraminidase activity level of Urabe AM9 mumps virus hemagglutinin-neuraminidase glycoprotein Microbes Infect 2007 9 2 234 240 10.1016/j.micinf.2006.11.011 17223599

[B20] Rosas-Murrieta N Herrera-Camacho I Vallejo-Ruiz V Millán-Pérez-Peña L Cruz C Tapia-Ramírez J Santos-López G Reyes-Leyva J Differential sensitivity to interferon influences the replication and transcription of Urabe AM9 mumps virus variants in nerve cells Microbes Infect 2007 9 7 864 872 10.1016/j.micinf.2007.03.005 17533145

[B21] Ulane CM Rodríguez JJ Parisien JP Horvath CM STAT3 ubiquitylation and degradation by mumps virus suppress cytokine and oncogene signaling J Virol 2003 77 11 6385 6393 10.1128/JVI.77.11.6385-6393.2003 12743296 PMC155014

[B22] Li T Chen X Garbutt KC Zhou P Zheng N Structure of DDB1 in complex with a paramyxovirus V protein: viral hijack of a propeller cluster in ubiquitin ligase Cell 2006 124 1 105 117 10.1016/j.cell.2005.10.033 16413485

[B23] Mao X Ren Z Parker G Sondermann H Pastorello M Wang W McMurray J Demeler B Darnell J Chen X Structural bases of unphosphorylated STAT1 association and receptor binding Mol Cell 2005 17 6 761 771 10.1016/j.molcel.2005.02.021 15780933

[B24] Chen X Vinkemeier U Zhao Y Jeruzalmi D Darnell JE Kuriyan J Crystal structure of a tyrosine phosphorylated STAT-1 dimer bound to DNA Cell 1998 93 5 827 839 10.1016/S0092-8674(00)81443-9 9630226

[B25] Young DF Chatziandreou N He B Goodbourn S Lamb RA Randall RE Single amino acid substitution in the v protein of simian virus 5 differentiates its ability to block interferon signaling in human and murine cells J Virol 2001 75 7 3363 3370 10.1128/JVI.75.7.3363-3370.2001 11238862 PMC114129

[B26] Puri M Lemon K Duprex WP Rima BK Horvath CM A point mutation, E95 D, in the mumps virus v protein disengages STAT3 targeting from STAT1 targeting J Virol 2009 83 13 6347 6356 10.1128/JVI.00596-09 19386700 PMC2698558

[B27] Young DF Didcock L Goodbourn S Randall RE Paramyxoviridade use distinct virus-specific mechanisms to circumvent the interferon response Virology 2000 269 2 383 390 10.1006/viro.2000.0240 10753717

[B28] Brown EG Dimock K Wright KE The Urabe AM9 mumps vaccine is a mixture of viruses differing at amino acid 335 of the hemagglutinin-neuraminidase gene with one form associated with disease J Infect Dis 1996 174 6 619 622 8769623 10.1093/infdis/174.3.619

[B29] Brown EG Wright KE Genetic studies on a mumps vaccine strain associated with meningitis Rev Med Virol 1998 8 3 129 142 10.1002/(SICI)1099-1654(199807/09)8:3<129::AID-RMV213>3.0.CO;2-Z 10398501

[B30] Yokosawa N Kubota T Fujii N Poor induction of interferon-induced 2',5'-oligoadenylate synthetase (2-5 AS) in cells persistently infected with mumps virus is caused by decrease of STAT-1a Arch Virol 1998 143 10 1985 1992 10.1007/s007050050434 9856085

[B31] Fujii N Yokosawa N Shirakawa S Suppression of interferon response gene expression in cells persistently infected with mumps virus, and restoration from its suppression by treatment with ribavirin Virus Res 1999 65 2 175 185 10.1016/S0168-1702(99)00114-8 10581390

[B32] Fagerlund R Melen K Kinnumen L Julkumen I Argine/lysine-rich NLSs mediate interactions between dimeric STATs and importin alpha 5 J Biol Chem 2002 277 33 30072 30078 10.1074/jbc.M202943200 12048190

[B33] Andrejeva J Young DF Goodbourn S Randall RE Degradation of STAT1 and STAT2 by the V proteins of simian virus 5 and human parainfluenza virus type 2, respectively: consequences for virus replication in the presence of alpha/beta and gamma interferons J Virol 2002 756 5 2159 2167 10.1128/jvi.76.5.2159-2167.2002 PMC153821 11836393

[B34] Didcock L Young DF Goodbourn F Randall RE The V protein of simian virus 5 inhibits interferon signalling by targeting STAT1 for proteasome-mediated degradation J Virol 1999 73 12 9928 9933 10559305 10.1128/jvi.73.12.9928-9933.1999 PMC113042

[B35] Arnold K Bordoli L Kopp J Schwede T The SWISS-MODEL Workspace: A web-based environment for protein structure homology modelling Bioinformatics 2006 22 2 195 201 10.1093/bioinformatics/bti770 16301204

[B36] Gibrat JF Madej T Bryant SH Surprising similarities in structure comparison Curr Opin Struct Biol 1996 6 3 377 385 10.1016/S0959-440X(96)80058-3 8804824

[B37] Guex N Peitsch MC SWISS-MODEL and the Swiss-PdbViewer: An environment for comparative protein modeling Electrophoresis 1997 18 15 2714 2723 10.1002/elps.1150181505 9504803

[B38] Laskowski RA MacArthur MW Moss DS Thornton JM PROCHECK: a program to check the stereochemical quality of protein structures J Appl Cryst 1993 26 283 291 10.1107/S0021889892009944

[B39] Wiederstein M Sippl MJ ProSA-web: interactive web service for the recognition of errors in three-dimensional structures of proteins Nucl Acid Res 2007 35 Suppl 2 W407 W410 10.1093/nar/gkm290 PMC1933241 17517781

[B40] Combet C Jambon M Deléage G Geourjon C Geno3D: automatic comparative molecular modelling of protein Bioinformatics 2002 18 1 213 214 10.1093/bioinformatics/18.1.213 11836238

[B41] Maiti R Van Domselaar GH Zhang H Wishart DS SuperPose: a simple server for sophisticated structural superposition Nucleic Acids Res 2004 32 Web Server W590 W594 10.1093/nar/gkh477 15215457 PMC441615

[B42] Tung CW Ho SY Computational identification of ubiquitylation sites from protein sequences BMC Bioinform 2008 9 310 324 10.1186/1471-2105-9-310 PMC2488362 18625080

[B43] Ritchie DW Kemp GJL Protein docking using spherical polar Fourier correlations Proteins 2000 39 2 178 194 10.1002/(SICI)1097-0134(20000501)39:2<178::AID-PROT8>3.0.CO;2-6 10737939

[B44] Schneidman-Duhovny D Inbar Y Nussinov R Wolfson HJ PatchDock and SymmDock: servers for rigid and symmetric docking Nucleic Acids Res 2005 33 Web Server W363 367 10.1093/nar/gki481 15980490 PMC1160241

[B45] Mashiach E Schneidman-Duhovny D Andrusier N Nussinov R Wolfson HJ FireDock: a web server for fast interaction refinement in molecular docking Nucleic Acids Res 2008 36 Web Server W229 232 10.1093/nar/gkn186 18424796 PMC2447790

